# Diseases in the Peninsula

**Published:** 1830-09

**Authors:** G. J. Guthrie

**Affiliations:** 2, Berkeley street


THE LONDON
### Medical and Physical Journal.

N° 379, VOL. LXIV.] SEPTEMBER 1830. [N° 51, *New Series.*

For many fortunate discoveries in medicine, and for the detection of numerous errors, the world is indebted to the rapid circulation of Monthly Journals; and there never existed any work to which the Faculty, in Europe and America, were under deeper obligations than to the *Medical and Physical Journal of London*, now forming a long but invaluable series.—RUSH.

### ORIGINAL PAPERS, AND CASES,

### OBTAINED FROM PUBLIC INSTITUTIONS AND OTHER AUTHENTIC SOURCES.

*To the Editor of the London Medical and Physical Journal.*

SIR: I have accidentally found, among some other manuscripts, a Report on the Causes of the Sickness which prevailed in the fourth Division of Infantry of the British Army, consisting of near 6000 men, in Spain and Portugal, during part of the years 1811 and 1812, which was sent by me to Sir JAMES M'GRIGOR, the inspector general of hospitals; dated St. Joâo de Pesquiera, May 1812. On some future occasion it may perhaps be of use, as a record of facts, in the preservation of human life: it will also bring to the recollection of some who served with me, the remembrance of many days of toil and misery; and it may help to remind others of labours which are nearly forgotten.

I am, sir, your obedient servant,

G. J. GUTHRIE.

*2, Berkeley street; August* 14, 1830.

### DISEASES IN THE PENINSULA.

THE diseases of Spain and Portugal, on the southern and western frontier, in summer and autumn, very much resemble those of hot climates in general, and, the nearer the approach to the south, the greater is the similarity; so much so, that I have frequently seen fevers at Gibraltar, Cadiz, and in the plains of the Guadiana, in every respect agreeing with the description given of the bilious remittent and yellow fevers of the West Indies and of the American states; as sudden and violent in their attack, as rapid in their progress, running the same train of symptoms, and leaving frequently the same results.

The difference of heat between the plains of Castile and of Estremadura is not very great: in the hot months of July, August, and September, the rivers and tracks of torrents from the neighbouring mountains are nearly dry; and they run generally in valleys or low grounds, which were marshes in winter and spring, and afford, in the hot and dry months, a large decomposition of vegetable matter, which is greatly assisted by the violent heat of the day and the great dews by night. The extreme heat also predisposes much to disease; of which the natives are so much aware, that they always, if possible, avoid exposing themselves to a midday sun; from whence arises their custom of dining at twelve, and taking their siesta, or sleeping for three or four hours afterwards, if their circumstances will permit; or they employ that time in various household concerns within doors. The British troops, on the contrary, never take the slightest precaution of the kind, or shelter themselves from the heat in their private occupations, and the necessities of a military life frequently demand their exposure; they, therefore, suffer much from the diseases prevalent in the country, or naturally predominating with the season of the year. In the wet and cold months of March and April, in the provinces of Portugal and of the western part of Spain, north of the Tagus, the obvious diseases are, synochus, inflammatory affections of the chest and limbs, and intermittent fevers for the most part amongst those who had suffered in the autumn. These continue with the warmer weather of May and June; but, in June, July, August, and September, the diseases more peculiar to warm climates show themselves with the excessive heat. The attack is more sudden, the excitement greater, and the determination to the head frequently excessive in the latter months. The effects of heat are combined with those of marsh effluvia, the fevers are remittent and intermittent: in many, the excitement, which is at first great, is soon, however, followed by extreme debility, general yellowness of skin, excessive irritability of the stomach, great discharges of vitiated bilious matters, petechiæ, glassy appearance of the eyes, dilatation of the pupil, coma, and death in a very few days: but this aggravation of disease is infrequent, or runs a longer course, to the north of the Tagus. The patient slowly recovers; has probably a paroxysm of intermittent from time to time, or only an occasional febrile uneasiness; does not recover the natural complexion; and, even when there is every appearance of convalescence, the least exertion will cause a relapse. In general, recovery is very protracted: frequently, after an interval of three and four weeks, the relapse would be sudden, and carry off the patient in a few hours; and in some instances the malady has been supposed to be apoplectic, yet the appearances on dissection did not warrant this conclusion. Dysentery, which in particular constitutions is also supposed to arise from marsh miasma, is not a formidable disease during the summer months, and even in the first weeks of October, after the commencement of the occasional rains, nor until the month of November, when, with the cold and damp weather, the attendant fever has become typhoid, with great debility: it is then exceedingly dangerous, inasmuch as the means of cure, so successfully employed when the fever is simple, continued, or even remittent, are contra-indicated by the extreme debility experienced in the cases of typhus not suffering a decided treatment to be enforced; the relief of the febrile symptoms, or means employed to obviate death from debility, frequently assisting in establishing a chronic state of dysentery, that eventually carries off the patient.

The fever, in the greater part of the admissions in the different regiments of the division, in the months of November, December, and January, was typhus mitior or gravior, depending much on the constitution of the patient. In the recruits and relapsed cases it was always the latter, and where the interval, or state of convalescence, had been unfavorable, was generally unfortunate. In the month of February, with the return of spring, some inflammatory affections of the chest appeared, but in certain constitutions the fever soon became typhoid, and the event fatal.

The officers during this period suffered equally with the men, particularly from June to Christmas. Intermittent and remittent fevers, in the worst forms, were observed in several, and considerable subsequent derangement of the abdominal viscera; but, as they were all young men, generally of good constitution, had the means of procuring good clothing, sufficiency of food, and shelter from the inclemency of the weather, during the siege of Ciudad Rodrigo, and in the subsequent month of January, few suffered from typhus, and none died.

The mortality amongst the inhabitants of the different villages, in the period alluded to, was greater than that of the troops, especially in the summer season, and in places where the soldiers were not constantly quartered; which may be accounted for from the wretchedness of their situation, and frequently from the want of appropriate remedies.

From the comparative history of warm climates, and from the experience of four years by the British army in Spain and Portugal, these diseases are known in autumn to be endemic, and that the natives suffer from them as well as the troops. They arise from the influence of excessive heat and marsh effluvia, and are to be avoided only by non-exposure to their exciting causes, more especially when the body is predisposed to disease. There are many circumstances exclusively connected with a military life that cause this predisposition, independent of the susceptibility natural to young persons in a foreign country, and in which their manner of living is perhaps totally altered. A combination of these causes has produced a disease so general amongst a certain number of people as to excite an idea of its being contagious, especially when it has occurred with troops that have been but a short time in the country. It is, however, now generally believed not to be communicable from one person to another, whilst it preserves the true remittent or continued form of fever arising from excessive heat and marsh effluvia. At a later period, when the influence of these causes has ceased, the body, on the application of other accidental exciting causes of fever, being in a state of predisposition for disease from the effects of former illness, is soon debilitated; the assemblage of symptoms denote what is called typhous fever, and a disease is formed capable of reproducing itself by communication, as has been well marked here in several instances. In 1809, when the army suffered so severely in the plains of the Guadiana from remittent fever in September and October, and from dysentery and typhus in the three subsequent months, I was in the habit of ordering two fresh orderlies daily to keep up an establishment of sixteen from one regiment only. Every hospital was in the same situation, and, in the present instance, at the same period of disease the troops suffered considerably, not only in men who had been some time with the sick, but in others who had come from some little distance in an apparent state of health.

During the spring and summer months of 1811, the fourth division was most actively employed, and much exposed to the wet and hot weather. In the month of August, during a march of sixteen miles from Frontera to Crato, the heat was excessive, and one day so much so as to cause 300 men to fall out of the ranks. In September, the division was cantoned in the villages in front of Almeida, in the vicinity of the Turon and Duas Casas rivers; and it would appear, the effects of former fatigue, and the influence of heat and marsh effluvia, were great, and particularly observable in the seventh regiment, which was quartered on each side of the river; 203 admissions of fever occurring, of which three died, and 122 were sent to the rear.

From the 20th of September to the 20th of October, this corps suffered much from fatigue, privation of food, heat, and the occasional rains, which fell very heavily. During the time they bivouaced, 200 fevers were admitted, one died in regimental hospital, and sixty-three were sent to the rear.

From the 20th of October to the 20th of November, 156 fevers were admitted; six died, twenty-six were sent to the rear.

From the 20th of November to the 20th of December, the division, on an advance to Ciudad Rodrigo, to prevent the entrance of a convoy, suffered much from every kind of hardship and privation; the weather being extremely cold, the easterly winds most piercing, and the troops hardly under cover, without proper clothing, and with a short allowance of food. Of the seventh regiment, 124 fevers were admitted; none died, thirty-two were sent to the rear.

To the 20th of January, 144 fevers were admitted; seventeen died, and twenty-three were sent to the rear. At this period the fevers were generally typhous, the debility great, and frequently those patients who relapsed from a former fever, after an intermediate state of convalescence, or who were almost in health, died in a day or two. In this month, during the siege, the division was much exposed, from the insufficiency of the quarters allotted to the troops, from the hard weather during the siege, and the heavy rains immediately following it.

To the 20th of February, 105 fevers were admitted; forty-one died, and eighteen were sent to the rear.

To the 20th of March, ninety-one fevers were admitted; nine died, and 158 were sent to the rear: the regimental establishment breaking up on the division marching to the south.

In the six months, there were 1329 admissions from all complaints: of these, 86 died in regimental hospital, and of which 75 were recruits; 427 were sent to general hospital, including 320 fevers, of which 160 died. Of the 246 dead in the six months, 169 were recruits, out of 353 landed at Lisbon in June 1811; 77 were old soldiers, out of 1145: that is, nearly half of the recruits, and one sixth of the old soldiers.

The fortieth regiment, nearly under the same local circumstances, and of the same strength, suffered in a proportion somewhat less; having 1189 sick admitted into the regimental hospital from the 20th of March unto the 20th of September: of these, 869 were fevers, of which 446 were sent to the rear. In that period, 24 died in regimental hospital, 156 in general hospital. The fortieth regiment received 400 men from England in July from the second battalion, and these also suffered infinitely more than the older soldiers, and from the same causes as the seventh regiment; but, being a better sort of men in general, the mortality was not so great. Of 450 recruits, 102 died; of 1117 old soldiers, seventy-eight died in the six months: that is, nearly a fourth of the recruits, and a fourteenth of the older soldiers.

The third battalion of the twenty-seventh, nearly composed of Irish and of young men, during this period remained remarkably healthy, possessing no advantage over the others in local situation until December, and being rather inferior in every respect to the seventh and fortieth regiment in point of regimental economy: most of these, however, had been two or three years in the country, and the drafts they received were Irish boys, more accustomed to hardships and less easily subdued by it. From the 20th of September to the 20th of March, there were admitted into the regimental hospital altogether 454 sick only; of which, 259 were sent to general hospital, eight died in regimental hospital, and fifty in general hospital.

In December, the twenty-seventh regiment, being nearly naked, were sent from the front to Coimbra, and remained away until the beginning of February, returning complete in every thing, but avoiding thereby the siege of Rodrigo, and the difficulties and hardships of the month of January; which, I think, had a great effect, both as to the prevalence of disease and the subsequent mortality.

From the 20th of September to the 1st of March, no man was sent from the division supposed to be in a dangerous state. On the 1st, the regimental establishments were broken up.

The state of the seventh regiment was always an object of concern and particular attention to the commanding officer, the general of division, its medical officers, and myself; and I believe every thing our means would permit were tried. They were, as much as possible, protected from the weather. Straw and hay mats were made, on every change of quarter, for the men to sleep on; their messes, and the cleanliness of their houses, were rigidly attended to; the night guards and duties were reduced as much as possible; they were never allowed to occupy or remain in a house with a sick inhabitant. The blankets, as they came up, were given to the convalescent men, in preference to the usual mode of so many per company. No man was put on duty unless perfectly strong. During the siege of Ciudad Rodrigo, near one hundred men, mostly recruits, who were doing duty with the regiment, were left in their old quarters, under proper officers, and an additional sum was drawn from their pay to procure them a better mess. The quartermaster general and myself frequently examined their quarters, and gave more room if we thought they were confined; and the general officers of brigade and division frequently inspected the hospitals.

Judging from these circumstances, and the superior attention paid to the seventh regiment, they should have been at least as healthy as others, where the same strictness was not observed; which was, however, not the case, probably from their suffering the whole time the more combined operation of all the causes of disease, and which, under the existing circumstances, could not readily be avoided.

A fertile source of disease in this country, and which might readily be prevented, arises from the paying off the whole balance due to the troops, whenever the money can be procured: thus furnishing them the means of being drunk for the greater part of three or four days, instead of purchasing daily or weekly such articles of subsistence as could be obtained, and which, in addition to the ration, would secure to them a breakfast in quarters. This fact is so notorious, that the surgeons, and even the officers who pay the money, generally consider an increase of sickness a matter of course.*

Disease is also greatly aggravated by the mode of conveyance from the regiments to the establishments in the rear. This is usually effected through the means of the commissariat, either by mules or bullock cars, in or upon which the sick are exposed to the heat, wet, and indeed all changes of weather. These conveyances even were not

* This was shortly afterwards put a stop to.

given as required, but as they could be spared: consequently, the sick can only be removed every three or four days, and, when the depot is six or seven leagues in the rear, it generally requires two nights on the road, (certainly with cars,) and, during these two days and nights, (independently of the exposure to the weather,) the attention paid to them medically, or to their accommodation at night in quarters, is but trifling; so that men who leave their regiment after an illness of a couple of days, especially in the autumn, during the prevalence of remittent fever, frequently die upon the road, or shortly after they reach the place of their destination. If they escape, their disease is, for the most part, certainly increased. But if each division had a competent conveyance of its own, the journey to the first hospital would be accomplished with one halt; and as, during the unhealthy season of the year, the troops are nearly stationary on one line of march, at that halting place the non-commissioned officer could secure a supply of straw or mats to keep them from the ground. The three spring waggons attached to the division are, from construction and other causes, independent of the insufficiency of their number, frequently useless, and are a greater expense than a well-arranged ambulance would be on different principles.

The regiments in Portugal are kept up by draughts from the battalions at home, and, in obedience to the orders on the subject, are as much as possible drafted by complete files; attention, I believe, being paid only to reject old men, or those who, from accident, are unfit for active service, and not much to the age and appearance of the young ones, in consequence of which many are sent to this country who are incapable of performing the duties of a soldier. This was very evident in the recruits, or draft, sent from the second battalion of the seventh regiment to the first, and who arrived at Lisbon in June, in number 353: of these, eighty-seven never joined the regiment, and the greater part of those that did join were totally unequal to the service required of them. They had embarked in summer, left Lisbon in August, and reached their regiment on the 26th of September at Fuente Guinaldo: having, therefore, been removed to a climate several degrees hotter than their own; marched three weeks with a heavy load they were unaccustomed to in the worst season of the year, and which they could not have done without injury even in England. The ration of a pound of meat, including bone, and a pound and a half of bread, or one pound of biscuit, is hardly enough for growing boys, if issued regularly; but, when three days' allowance is given at once, it is in most instances eaten by the morning of the third day; so that, until the next issue, which may not be until the evening of the next day, they are without food. The recruits of the seventh suffered much from this cause; and they unfortunately arrived at a time (during the movement from Guinaldo to Sabugal,) in which provisions were extremely scarce, and issued in small quantity; they had a great deal of fatigue, and lay out without blankets during some wet weather. Their bodies were naturally at this moment predisposed to disease; in many it was already formed; and the subsequent season of the year being most unfavorable to such subjects, it is reasonable to suppose they must suffer accordingly. In fact, they never did recover the injury they had sustained, were most miserable objects, and were as readily distinguished from the older soldiers as if they had belonged to another regiment.

The great heat in summer renders it desirable to load the soldier as little as possible: the blanket, or great coat, is therefore taken away; hitherto generally the former, to avoid losing the coat, which is only replaced every three years, whereas new blankets are issued yearly, if they can be procured; but, from the difficulty of conveyance to the army on the frontier, the troops do not get them at a proper time. In my opinion, a soldier should be complete with blanket and coat by the 1st of October, to protect him from the cold and damps of the night, which are so great in a warm climate. The seventh regiment had not more than one blanket between two men in December, and were not complete in January; so that relapses could not fail to occur from that cause alone, for little assistance could be procured by the soldiers from the inhabitants; and occasionally, in fact twice during the winter, from the incidents of the war, the little comforts they could procure were for a time taken away, rendering them, of course, more susceptible when exposed.*

From a consideration of several drafts sent to this country, and particularly the inutility of the greater part of the 353 men sent in June to the seventh regiment, I would suggest the necessity of selecting men who have been some little time habituated to the life of a soldier, for the drafts to Spain and Portugal: or, if the want of men will not allow that to be done, of not sending out boys of seventeen and eighteen, especially if English, that have been brought up

* Tents were subsequently supplied.

in manufacturing towns, as they are generally of weakly constitutions, have been little accustomed to hardship, do not readily bear them, and are, in fact, but a nominal increase of strength. The men chosen for this service should be embarked in October, in large transports or men of war, these being always the most healthy, from the greater facility afforded the officers in attending to the men, from their having a larger space to move about in, even with an equal proportion of tonnage, and from their always having fresh air in bad weather, the hatches being open, and a number of men upon deck ; which cannot be suffered in small brigs of 150 or 180 tons burden, in which, with every care, they are generally wet and much confined. On their arrival at Lisbon in November, I would recommend their being quartered in proper places in the vicinity, or within thirty miles, for three or four months, until they become a little accustomed to the mode of life they had to undergo, and which is very different from that of a soldier at home. They should be regularly messed twice a day, and examined daily in fine weather, in marching order. In April and May, by short marches, and frequent halts of two and three days, they might join their regiment; the same care being taken of them as to a meal of some kind in the morning, when possible. They would now have had a six months' trial, and would be better able to support the privations and hardships they must experience during the campaign. In October their clothing should be completed, the days then being frequently hot and dry, the nights cold and damp; from the influence of which they should be carefully guarded. If this or some other cautious method were adopted, I think the health of the army would be much improved, and very many lives saved. In the instance of the seventh regiment, I have little doubt one half, or perhaps 200 of the original number, would have been effective under the plan proposed, instead of forty-two, the number now present with the regiment.

The climate of Portugal is too much valued in England for qualities it does not possess : indeed, it appears to have been little known beyond the neighbourhood of Lisbon, until within these few years. The advantages generally enumerated are taken from the vicinity of the capital, and these are very much overrated. The whole of the east of Portugal, and the parts occupied by the British army north of the Tagus, are more unhealthy than any part of England. In July, August, and September, this occurs from the intense heat, and the evaporation and the decomposition of vegetable matter in the marshy grounds and courses of rivers: in November, December, January, February, and March, from the heavy rains, cold winds, and frequent changes of weather, which are particularly felt from the houses in general, and especially on the frontier, being badly built, possessing neither chimneys nor windows, by the aid of which an equal degree of temperature might be supported. The unhealthiness of September and October is proverbial in Estremadura. April is very changeable: so that, in fact, May and June are the only favorable months for invalids; and the latter month is frequently very hot. Upon the whole, the climate of this part of Portugal is almost always unfavorable for the reestablishment of health after fevers; is particularly so after chronic or visceral diseases; and, from its frequent and great vicissitudes of weather for six months in the year, with the impossibility of counteracting them by artificial means, is adverse to pulmonic and rheumatic complaints.

Neither phthisis nor scrofula are prevailing diseases in Portugal, particularly in the neighbourhood of Lisbon. Cases of both do occasionally occur, but they are by no means so frequent as to render them more than accidental, which shows the little predisposition of the Portugueze for them. They are, however, during the great heat in summer, liable to hæmoptysis, under any violent exertion, but which is usually subdued by mild treatment, and the proper functions of the lungs restored without much difficulty. The constitution and mode of living of the Portugueze do not dispose them to suffer from inflammatory diseases; and the greater clearness of the atmosphere, and the equality of the seasons, together with the want of tone of the whole system, render inflammatory affections of the lungs less violent, and more easily cured, than in England. There is also little or no hereditary predisposition for pulmonary disease among them.

From such information as I can collect from my acquaintances in civil life, as well as from my own observation, it would appear that persons visiting Portugal from colder climates, with only a predisposition for phthisis, or with tubercles in a nearly quiescent state, or not in that of suppuration, do receive some benefit from the sea voyage and change of air; but, when suppuration and ulceration have taken place, which is the case in most persons sent from England, the result is almost always fatal in as short a time as if the experiment of change of climate had not been tried. I now know several cases deteriorating as fast, during the last two months, as I conceive they would have done, with due caution, at home. Chronic coughs are always benefited by a residence in this country.

